# Proliferative Activity (Ki-67 Expression) and Outcome in High Grade Osteosarcoma: A Study of 27 Cases

**DOI:** 10.1155/S1357714X00000086

**Published:** 2000-03

**Authors:** Roland Jong, Aileen M. Davis, Maria G. Mendes, Jay S. Wunder, Robert S. Bell, Rita Kandel

**Affiliations:** 1 Department of Pathology and Laboratory Medicine Mount Sinai Hospital University of Toronto Toronto Ontario M5G 1X5 Canada; 2 University Musculoskeletal Oncology Unit Mount Sinai Hospital University of Toronto Toronto Ontario M5G 1X5 Canada

## Abstract

*Purpose.* Although pre-operative chemotherapy has improved the prognosis for individuals with osteosarcoma, approximately 40% of patients will die of their disease.The aim of this study was to quantitate proliferative activity in high grade osteosarcomas and to determine whether proliferation is a prognostic factor.

*Patients.* The study consisted of 27 patients with high grade non-metastatic osteosarcoma at various sites for whom pre-operative biopsies and resection specimens were available for review. All patients were treated similarly and had at least 24 months' follow-up from the date of diagnosis.

*Methods.* Proliferative activity (Ki-67 expression) was examined in the diagnostic biopsies immunohistochemically using the MIB-1 antibody. Proliferation was quantitated in two ways; (1) the number of immunopositive cells was counted manually using an ocular grid; or (2) the percentage of immunopositive nuclear area was assessed using morphometric image analysis. Proliferative index was evaluated in relation to patient outcome.

*Results.* Proliferative activity was seen in all biopsies.The median proliferative index as determined by counting cells was 24% (mean of 27%, range of 7–61%) and by image analysis was 2% (mean 3%, range 0.32–8.4).The correlation between MIB-1 proliferation indices determined either by image analysis methodology or manual cell counting was high (Spearman's rho=0.79). Proliferative index did not appear to predict either disease-free or overall survival.

*Discussion.* Tumor proliferation does not appear to be prognostic for high grade osteosarcomas.Whether assessment of this feature in conjunction with other tumor characteristics might be prognostic requires further study.


*Sarcoma* (2000) **4**, 47–55

ORIGINAL ARTICLE

### Proliferative activity (Ki-67 expression) and outcome in high grade osteosarcoma: a study of 27 cases

ROLAND JONG,[1] AILEEN M. DAVIS,[2] MARIA G. MENDES,[1] JAY S. WUNDER,[2] ROBERT S. BELL,[2] & RITA KANDEL[1]

[1]*Department of Pathology and Laboratory Medicine and* [2]*University Musculoskeletal Oncology Unit, Mount Sinai Hospital, University of Toronto, Toronto, Ontario M5G 1X5, Canada*

**Abstract**
*Purpose.* Although pre-operative chemotherapy has improved the prognosis for individuals with osteosarcoma, approximately 40% of patients will die of their disease. The aim of this study was to quantitate proliferative activity in high grade osteosarcomas and to determine whether proliferation is a prognostic factor.
*Patients.* The study consisted of 27 patients with high grade non-metastatic osteosarcoma at various sites for whom pre-operative biopsies and resection specimens were available for review. All patients were treated similarly and had at least 24 months' follow-up from the date of diagnosis.
*Methods.* Proliferative activity (Ki-67 expression) was examined in the diagnostic biopsies immunohistochemically using the MIB-1 antibody. Proliferation was quantitated in two ways; (1) the number of immunopositive cells was counted manually using an ocular grid; or (2) the percentage of immunopositive nuclear area was assessed using morphometric image analysis. Proliferative index was evaluated in relation to patient outcome.
*Results.* Proliferative activity was seen in all biopsies. The median proliferative index as determined by counting cells was 24% (mean of 27%, range of 7–61%) and by image analysis was 2% (mean 3%, range 0.32–8.4). The correlation between MIB-1 proliferation indices determined either by image analysis methodology or manual cell counting was high (Spearman's rho=0.79). Proliferative index did not appear to predict either disease-free or overall survival.
*Discussion.* Tumor proliferation does not appear to be prognostic for high grade osteosarcomas. Whether assessment of this feature in conjunction with other tumor characteristics might be prognostic requires further study.

**Key words**: *cell proliferation, MIB-1, osteosarcoma, prognosis*

With the addition of pre-operative chemotherapy to the treatment regimen for osteosarcoma the 5 year survival rates have increased, although approximately 30–40% of patients still succumb to the disease.[1,2] The current challenge is to delineate those patients who have a worse prognosis. It is well accepted that tumor grade influences prognosis as low grade osteosarcomas have little metastatic potential;[3] however, the identification of significant prognostic indicators for high grade osteosarcoma has been elusive. Although several studies have suggested that a variety of factors such as age, sex, tumor size and site at the time of presentation[2,4] may be of prognostic value, a critical analysis of the literature demonstrated that only tumor necrosis after chemotherapy has predictive value.[5] Patients whose tumors after chemotherapy show greater than 90% tumor necrosis in the resected specimen histologically had a continuous disease-free survival of 91%, as compared to 14% for those with tumoral necrosis of less than 90%.[6] However, this parameter cannot be used to predict those who will respond to chemotherapy as necrosis is only determined after chemotherapy has been given.

There is an increasing body of literature demonstrating that certain chemotherapeutic agents, such as cis-platin which is used in the treatment of osteosarcoma, act by inducing cellular apoptosis rather than having a direct toxic action on the cells.[7–13] Apoptosis follows a regulated pathway and allows for the elimination of abnormal or genetically damaged cells.[14,15] As programmed cell death occurs predominately in proliferating cells, we postulated that osteosarcomas with a high proliferative rate would be more sensitive to chemotherapy and that assessing proliferation in the tumor pre-operatively might be a way to predict prognosis. In keeping with this hypothesis it has been shown that an increased proliferative index is prognostic for a wide variety of tumors.[16–20]

In this retrospective study, proliferation was

Correspondence to: Dr. Rita Kandel, Department of Pathology and Laboratory Medicine, Mount Sinai Hospital, Room 600, 600 University Avenue, Toronto, Ontario M5G 1X5, Canada. Tel: (+1) 416-586-8516; Fax: (+1) 416-586-8268; E-mail: rkandel@mtsinai.on.ca

1357-714X print/1369-1643 online/00/010047-09 © 2000 Taylor & Francis Ltd examined in paraffin sections of diagnostic biopsies of osteosarcomas by immunostaining for Ki-67 expression using the MIB-1 antibody. The proliferation index was quantitated in two ways: (1) manual cell counting using an ocular grid; or (2) by determining the percentage of immunopositive nuclear area using morphometric image analysis. Proliferative activity was then evaluated in relation to the clinical outcome.

### Materials and methods

### *Clinical and tissue information*

Between 1989 and 1996, 27 patients with nonmetastatic osteosarcoma, for whom both biopsy and tumor resection specimens and clinical follow-up were available, were retrieved from the files of Mount Sinai Hospital. Low grade or surface osteosarcomas, osteosarcomas of the jaw or soft tissue, and osteosarcomas arising in pre-existing bone disease such as Paget's disease were excluded. As well patients whose biopsies had been decalcified prior to paraffin-embedding were excluded from the study as this can affect immunoreactivity.[21] All patients received three courses of chemotherapy which included adriamycin (75 mg/m$^2$) for 3 days and cis-platin (100 mg/m$^2$) prior to resection of the tumor and three courses of chemotherapy post-operatively. The minimum length of follow-up was 2 years from diagnosis.

The biopsies were fixed in 10% buffered formalin, and paraffin-embedded. Histological sections were stained with hematoxylin and eosin and examined microscopically. The tumor resection specimens were serially cut using a band saw, and X-rayed. Representative tumor sections, selected after review of the specimen X-ray and the gross appearance and representing approximately 30–50% of the tumor, were submitted for histological examination. The tissue was fixed in formalin for at least 24 hours, decalcified in 10% formic acid, and then paraffin-embedded. Five micron sections were cut and stained with hematoxylin and eosin. The percent of tumor which had undergone necrosis was estimated histologically in each slide which contained tumor.

### *Immunohistochemistry*

A representative section from each biopsy was dewaxed, microwave pretreated (2×5 min in antigen retrieval solution; Vector, Burlingame, CA), and blocked with a universal blocking agent (Signet Laboratories, Inc., Dedham, MA). The sections were incubated with antibody reactive with Ki-67 (MIB-1 at 1:50 dilution; AMAC Inc., Westbrook, ME) overnight. The negative control included leaving out the primary antibody. Immunoreactivity was detected using a biotinylated secondary antibody and an avidin–biotin peroxidase complex detection system (Vectastain, Vector, Burlingame, CA) using the chromagen 3-3'-diaminobenzidine tetrahydrochloride (Sigma Chemical Co., St. Louis, MI). The sections were counterstained with hematoxylin. In order to diminish the subjectivity of scoring, a brown nuclear reaction product of any intensity was considered a positive reaction.

### *Assessment of proliferative index*

*Cell counts.* The sections were scanned at low power, the areas in the tumor with the highest proliferative activity were selected and the MIB-1 labeled cells were counted using an ocular grid at 250× magnification. Three hundred tumor cells were counted, and if there were not 300 cells then all the tumor cells in the section were counted. All sections were counted at least twice by two observers who had no knowledge of the clinical outcome. When there was a discrepancy (>5% difference) between the counts, the two observers examined the slide under a multi-headed microscope and recounted the slide. The proliferative index was calculated as percent of the positive MIB-1 stained nuclei over the total number of tumor cell nuclei counted and the final value was the mean of the two readings.

*Image analysis.* The immunostained tissue sections were analyzed by light microscopy, with a 40× objective (400× magnification), and the Quantimet 500 MC image analysis system equipped with a color camera (Leica, Canada) by one observer (R.J.) who had no knowledge of the clinical outcome. Areas composed predominantly of tumor were selected and areas of reactive bone and fibrosis, hemorrhage and inflammation were preferentially excluded. Thirty consecutive fields (0.026 mm$^2$/field) were analyzed, starting from the lower left hand corner of each slide. The total positive stained area for each field was thresholded and a series of segmentation and fill commands were made manually using the edit command. The total positive area was related to the total field area measured. The percentage positive nuclear staining for each field was calculated by the Quantimet 500 MC system as the percentage of immunopositive nuclear area over the total field area. The intraobserver variability was on average 12% (data not shown).

*Statistical analysis.* Descriptive statistics including means, standard deviations and frequencies were calculated for sample descriptors and proliferation as appropriate. As proliferation was calculated using two methods, Spearman's rho was calculated to evaluate the relationship between the results. Similarly, the correlation of proliferation to tumor necrosis after pre-operative chemotherapy was evaluated. Proliferation was then dichotomized at the median value (24%) and compared to tumor necrosis dichotomized at 90% and then 95%. Proliferation as a predictor of

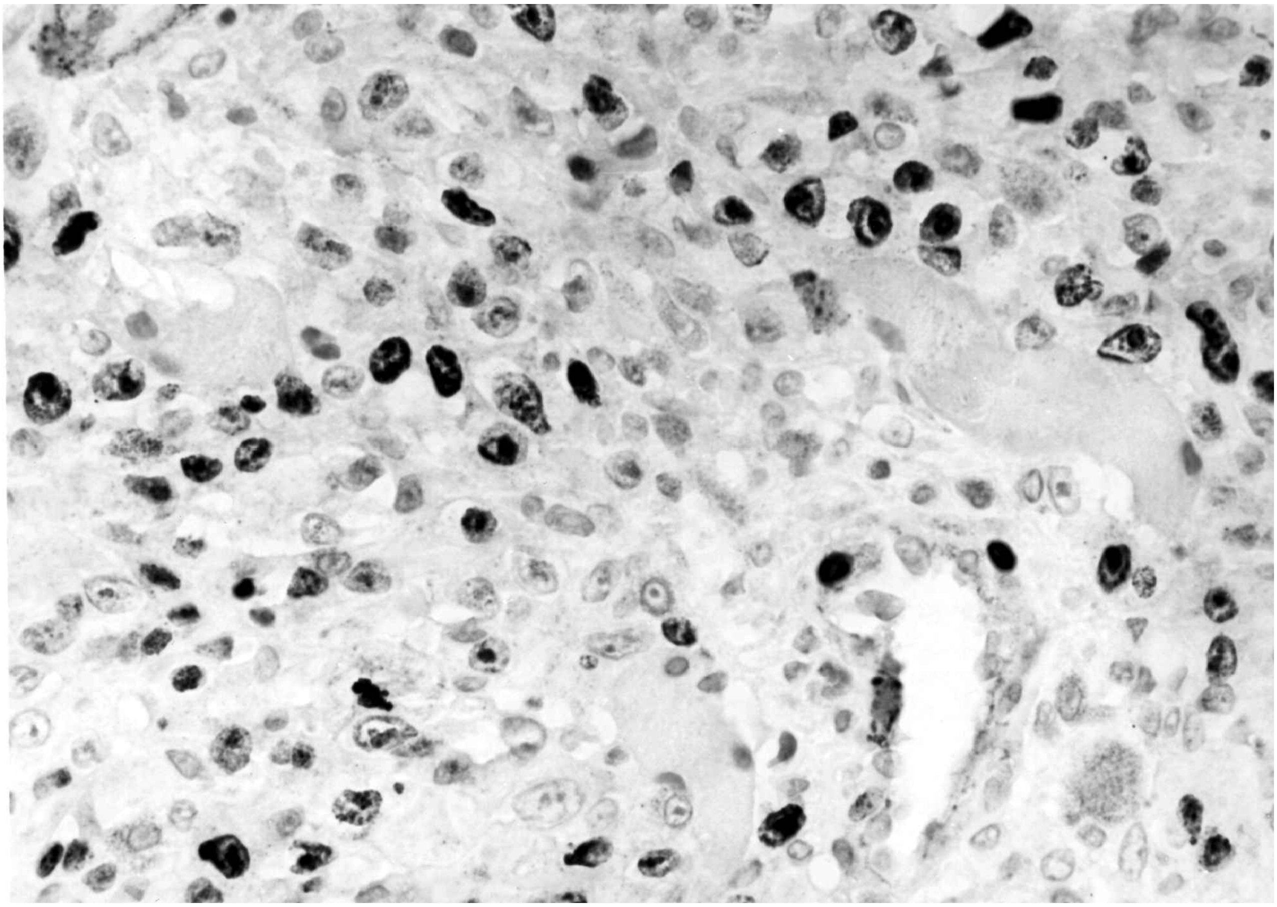

**Figure 1**. *Photomicrograph of osteosarcoma showing Ki-67 immunoreactivity as indicated by dark nuclear staining (immunoperoxidase, hematoxylin counterstain, magnification × 400).*

disease-free survival and overall survival was evaluated using the method of Kaplan and Meier[22] with the date of diagnosis as time zero.

### Results

### *Patient characteristics*

As shown in Table 1, the patients consisted of 17 males and 10 females with a median age of 20 years (range 14–64 years). The sites of involement included femur ($n$=12), tibia ($n$=3), humerus ($n$=1), radius ($n$=2), fibula ($n$=5), and pelvis ($n$=4). The mean maximal diameter of the tumor was 9.6 cm (range 4–17 cm). There were 4 stage IIA, and 23 stage IIB tumors. There were no obvious differences between stage IIA or IIB tumors in terms of tumor size or clinical course suggesting they could be analyzed together as one group. Eleven of the 27 patients (41%) relapsed systemically. The median disease-free survival for the entire group from the time of biopsy was 34 months (range from 4–93 months) (Table 2). The median overall survival was 39 months (range from 6–93 months) (Table 2).

### *Pathology*

Review of the biopsies demonstrated the presence of osteosarcoma as characterized by the presence of malignant cells producing osteoid.[3] The subtypes of osteosarcoma included osteoblastic, chondroblastic, fibroblastic, giant cell rich and small cell subtypes. The biopsies showed variable amounts of necrosis.

Residual osteosarcoma was seen in all but three of the resected tumors. When single cells with enlarged hyperchromatic smudged nuclei were seen these were interpreted as residual tumor cells. The tumors showed a variety of changes including overt necrosis, or tumor cell drop out with the pre-existing matrix remaining. In areas, the tumor had been replaced with fibrovascular tissue with or without ossification and these were considered reparative changes indicative of remote tumor necrosis and were included in the determination of tumor necrosis. The estimated amount of necrosis in the resected specimens varied between 5 and 100% (Table 2). Twelve of the 27 patients had greater than 90% tumor necrosis with 4 patients having greater than 95% tumor necrosis.

### *Ki-67 labeling index*

Proliferative activity, as indicated by Ki-67 (MIB-1) expression detected by immunohistochemical staining, was seen in all biopsies. The median proliferative index, as determined by manual counting was 24% (mean of 27%, range of 7–61%) and by image analysis was 2% (mean 3%, range 0.32–8.4). The proliferative index as determined by manual counts

**Table 1**. *Clinical features*

| Case | Age (yrs) | Gender | Site | Tumor size (cm)* | Stage† |
|---|---|---|---|---|---|
| 1 | 32 | male | femur | 4.9 | IIA |
| 2 | 23 | male | tibia | 6.8 | IIB |
| 3 | 20 | female | pelvis | 14 | IIB |
| 4 | 14 | female | radius | 7.5 | IIB |
| 5 | 29 | female | tibia | 9 | IIB |
| 6 | 21 | male | radius | 8.5 | IIB |
| 7 | 16 | male | humerus | 8 | IIB |
| 8 | 18 | female | fibula | 6.5 | IIB |
| 9 | 31 | male | femur | 6.5 | IIA |
| 10 | 15 | female | femur | 4.7 | IIB |
| 11 | 47 | male | femur | 7.5 | IIA |
| 12 | 20 | female | fibula | 4 | IIB |
| 13 | 31 | female | femur | 10.2 | IIB |
| 14 | 21 | male | femur | 15 | IIA |
| 15 | 49 | male | pelvis | 9 | IIB |
| 16 | 22 | male | tibia | 6 | IIB |
| 17 | 18 | male | femur | 10 | IIB |
| 18 | 20 | male | femur | 10.5 | IIB |
| 19 | 26 | female | pelvis | 16 | IIB |
| 20 | 17 | male | femur | 7 | IIB |
| 21 | 19 | male | fibula | 9.5 | IIB |
| 22 | 15 | female | fibula | 14.7 | IIB |
| 23 | 19 | male | femur | 17 | IIB |
| 24 | 20 | female | fibula | 4 | IIB |
| 25 | 64 | male | femur | 8 | IIB |
| 26 | 46 | male | pelvis | 17 | IIB |
| 27 | 17 | male | femur | 17 | IIB |

*Tumor size based on maximum diameter.
†Stage is determined according to AJCC criteria.[57]

showed a significant correlation with the proliferative index as determined by morphometric quantification of the percentage of Ki-67 positive nuclear area (Spearman's rho=0.79, $p<0.001$).

### *Correlation of Ki-67 labeling index with tumor necrosis*

The correlation of proliferation, whether by manual counting or morphometric quantification, with tumor necrosis was not significantly different than 0 (rho=0.25, $p=0.21$ and rho=0.23, $p=0.25$, respectively).

### *Ki-67 labeling index as a predictor of disease outcome*

As the two methods of quantifying proliferation were highly correlated, manual counting was arbitrarily chosen to evaluate the relationship of proliferation to disease-free and overall survival. Figure 2 shows that there was overlap in the proliferation values based on whether the patient developed systemic disease or not. Similarly, the time to systemic relapse ($p=0.90$) and overall survival ($p=0.54$) were not significantly different when proliferation was dichotomized at the median of the manual count, that is <24% or ≥24% (Fig. 3). Five of 13 patients with tumors showing <24% proliferation relapsed as compared to 6 of 14 patients with tumors showing ≥24% proliferation. As of last follow-up 5 patients had died of their disease (3 of 13 with proliferation <24% and 2 of 14 with proliferation ≥24%).

### Discussion

This study demonstrated that either manual cell counting or image morphometry can be used to determine proliferative index. However, the proliferation index, quantified in pretreatment biopsies of high grade osteosarcoma following immunohistochemical staining with the MIB-1 antibody, was not a prognostic indicator for either disease-free survival or overall survival. The follow-up time of 2 years which was used in this study is adequate to assess disease-free and overall survival as systemic metastases will develop in up to 94% of patients within 18 months of surgery.[6]

Two different methods were used to determine tumor proliferation; one was the standard method of counting cells using an ocular grid and the other utilized image analysis to quantify immunoreactivity by measuring the percent of immunopositive nuclear area. This latter approach has been utilized to predict prognosis in other tumor types.[19,23–25] Both methods of quantification were done to ensure that the way of determining cell proliferation was not influencing the results. There was a high correlation (rho=0.79) between the manual counting of the fraction of MIB-1 positive cells and the percentage area of MIB-1 nuclear positivity as measured by image analysis. This is in keeping with the report by Caulet *et al.*[26] who demonstrated in non-Hodgkin's lymphoma a similar correlation between proliferative indices determined by cell counting and by image analysis. It is unlikely

**Table 2.** *Proliferative indices and clinical outcome*

| Case | % area | % immunopositive cells | Tumor necrosis (%) | Systemic relapse (mos)* | Overall survival (mos)† |
|---|---|---|---|---|---|
| 1 | 0.32 | 17 | 50 | no | ANED (83) |
| 2 | 0.5 | 19 | 50 | no | ANED (41) |
| 3 | 0.58 | 12 | 75 | no | ANED (87) |
| 4 | 0.82 | 11 | 100 | no | ANED (37) |
| 5 | 0.97 | 15 | 70 | yes (4) | DOD (16) |
| 6 | 0.97 | 20 | 80 | yes (20) | ANED (30) |
| 7 | 1.04 | 11 | 30 | yes (10) | ANED (82) |
| 8 | 1.23 | 31 | 90 | no | ANED (27) |
| 9 | 1.25 | 7 | 40 | yes (6) | DOD (23) |
| 10 | 1.28 | 13 | 5 | no | ANED (56) |
| 11 | 1.66 | 34 | 90 | no | ANED (86) |
| 12 | 1.87 | 15 | 100 | no | ANED (34) |
| 13 | 1.95 | 20 | 65 | yes (8) | DOD (17) |
| 14 | 2.04 | 23 | 90 | no | ANED (41) |
| 15 | 2.49 | 13 | 90 | no | ANED (26) |
| 16 | 2.67 | 39 | 90 | yes (24) | DOD (42) |
| 17 | 3.07 | 27 | 95 | no | ANED (35) |
| 18 | 3.53 | 24 | 80 | yes (17) | AWED (38) |
| 19 | 3.82 | 28 | 75 | no | ANED (37) |
| 20 | 4.33 | 38 | 90 | no | ANED (44) |
| 21 | 4.44 | 41 | 50 | yes (25) | AWED (39) |
| 22 | 5.04 | 32 | 60 | yes (32) | AWED (40) |
| 23 | 6.24 | 30 | 90 | yes (4) | DOD (6) |
| 24 | 6.37 | 61 | 80 | no | ANED (69) |
| 25 | 7.24 | 47 | 40 | no | ANED (93) |
| 26 | 7.25 | 52 | 100 | yes (10) | AWED (25) |
| 27 | 8.4 | 60 | 90 | no | ANED (88) |

ANED = alive no evidence of disease; AWED = alive with disease; DOD = deceased due to disease.
* Months to relapse.
†Months of follow-up.
The proliferative indices in the biopsies of osteosarcoma, as determined by manual cell counts (% immunopositive cells) or image analysis (% area) were related to the amount of necrosis in the resected specimen and clinical outcome.

that the number of fields assessed by image analysis in the quantification of the proliferating index was a confounding factor. In a pilot study, we observed that when up to 50 fields were assessed, the percent positive nuclear area and the cell counts both leveled off between 25 to 30 fields (data not shown). Therefore, 30 fields were used for quantification using image analysis.

In keeping with lack of relationship between proliferative index and outcome, there was no relationship between proliferation index and tumor necrosis of greater than 90%. This is expected as this extent of necrosis has been shown to be predictive of outcome.[6]

Proliferative index was determined using MIB-1[27] and immunohistochemistry because the antibody (MIB-1) can be used on paraffin-embedded tissue and its reactivity is not affected if there is a delay in fixation.[28–30] The use of immunohistochemistry allows visualization of the immunoreactivity and confirms that it is located in tumor cells. Furthermore, proliferative indexes, determined using this approach, have been found to be prognostic in many different tumor types such as carcinoma,[20,31,32] lymphoma,[33] sarcoma[16–18,34] and brain tumors.[35] Proliferating cell nuclear antigen (PCNA) expression, a different proliferation associated protein, has been used by others to quantify proliferation. However, PCNA can still be detected 48 hours after entry into $G_0$ of the cell cycle[28] and PCNA is involved in DNA repair so the antibody can label nonproliferating cells,[28,36] both features limit its usefulness as a proliferation marker. There are at least four other methods to identify proliferating cells, counting mitoses, flow cytometry, nuclear organizer region (NOR) counts and DNA labeling, but they also have drawbacks. Mitotic count has been used but may underestimate the number of proliferating cells.[37–39] Flow cytometry does not detect cells in $G_1$,[38,40] DNA distributions can be difficult to interpret in aneuploid tumors,[30] and cellular debris and nontumor cells can be confounding factors.[29,30,41] Ag NORs (silver stained NOR) counts are another method to identify proliferating cells but they also appear in nonproliferating cells.[42] Ag NORs counts do not appear to estimate cell proliferation as well as Ki-67 immunostaining[43] and have been shown not to be prognostic in osteosarcoma.[44] DNA labeling by $^3$H-thymidine or BrDU labeling cannot be used in retrospective studies as the tissue has been formalin fixed.[28]

There have been four other studies[45–48] examining the relationship between proliferation index and osteosarcomas, and two of these showed results similar to the current investigation. None of these studies used image analysis for quantification. Park *et al.*[45] used

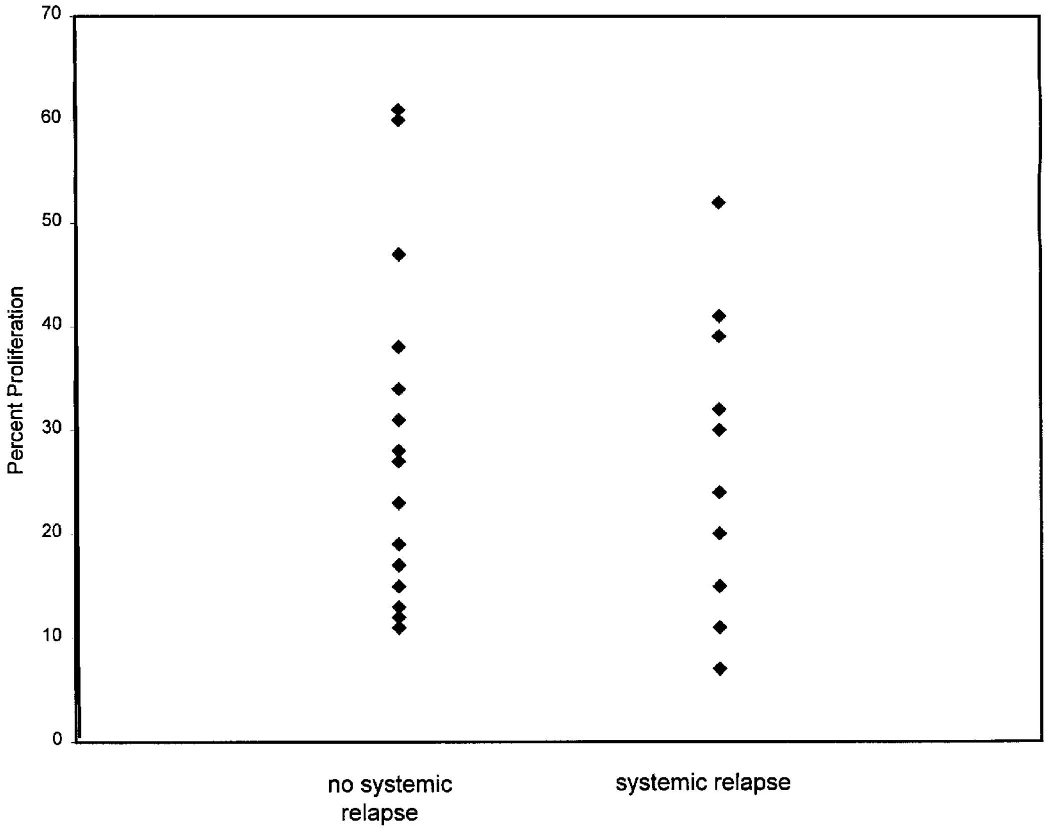

**Figure 2.** *Distribution of proliferative indices, as determined by manual cell counting, according to patients with no systemic relapse and those who had systemic relapse.*

PCNA and MIB-1 antibodies to assess proliferation in 34 cases of osteosarcoma and showed that proliferation was not predictive of disease-free survival. However, in that paper, it does not state whether the patients were all treated similarly. In the study by Scotlandi *et al.*,[46] the proliferative index was determined by measuring Ki-67 immunofluorescence in cytospins of 30 cases of high grade nonmetastatic osteosarcoma, which is a different method than that used in the current study. Although patients whose tumors had an elevated Ki-67 labeling index showed a trend ($p$=0.15) towards shorter disease-free survival at 24 months follow-up, it was not statistically significant, similar to our study. In contrast, the report by Molendini *et al.*[47] showed that patients with high grade osteosarcoma and a Ki-67 labeling index of ≥25%, which was the median value in that study, was prognostic as these patients had a statistically significant shorter disease-free survival than those with a value of less than 25%. However, this significance was lost in multivariate analysis. In that study the labeling index was determined by estimating the percentage of positive cells which only provides an approximate value. Furthermore, it was not stated whether these patients were treated in a uniform way suggesting that the study is not directly comparable to ours. In the fourth study,[48] although significantly longer survival was observed in osteosarcomas with a proliferative index less than 24%, the cases examined were extraskeletal osteosarcomas and they may behave differently than osteosarcomas of bone.

There are several possible explanations as to why proliferative index is not prognostic for osteosarcoma. First, the biopsy is only a small sample of the entire tumor and the proliferative fraction in the tissue obtained for biopsy may not be representative of the entire tumor. This is a less likely explanation as proliferative indices quantified in biopsies have been shown to be predictive of disease-free survival[49] or overall survival[50] in other tumor types. Second, although Ki-67 has been used extensively to assess proliferation in many tumor types, a recent study has shown that Ki-67 may not be a reliable marker of actively proliferating cells in osteosarcomas that are expressing p53 or the cell cycle protein p21.[51] Third, Ki-67 represents only one component of the cell cycle and it is possible that changes in other cell cycle proteins may also have to be considered as $G_1$ phase can be dysregulated in osteosarcoma.[52] For example, it has been shown that loss of cyclin D1 expression in osteosarcoma correlates strongly with metastatic progression and disease-free survival.[47] Fourth, the mechanisms regulating apoptosis may contribute to outcome.[13] Wild type p53, which regulates progression to apoptosis, when mutated can result in cellular resistance to chemotherapy.[12] Lastly, the mechanisms causing tumor resistance to chemotherapy are complex, and likely involve processes in addition to cell proliferation. Expression of multi-drug resistance proteins, such as mdr-1 or mrp, which limit intracellular accumulation of various chemotherapeutic agents are present in osteosarcoma cells[53–56]

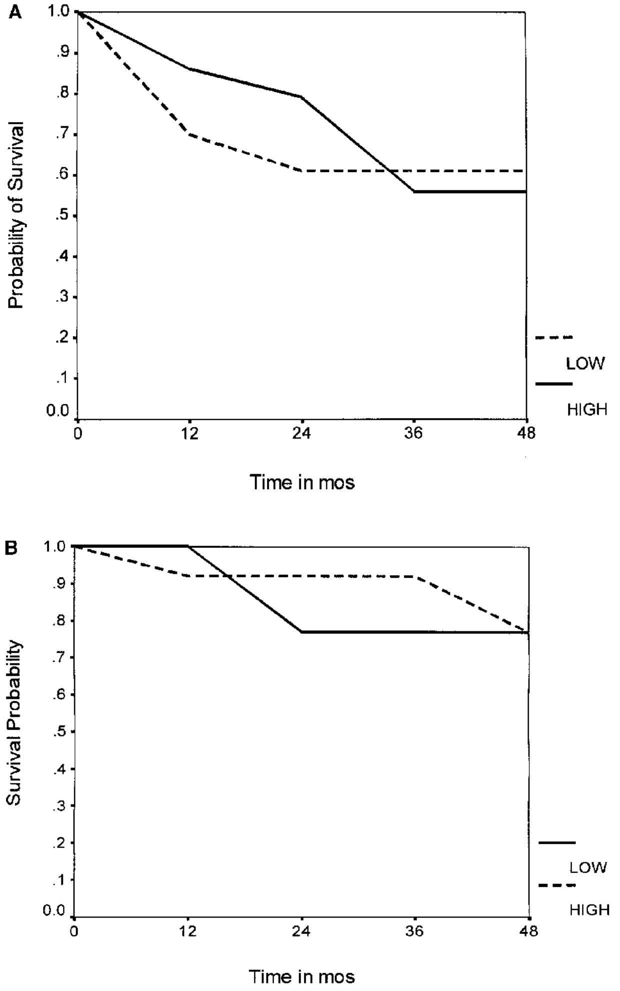

**Figure 3**. *(a) Kaplan–Meier curve showing disease-free survival relative to low proliferative index (<24%) or high (≥24%) proliferative index. (b) Kaplan–Meier curve showing overall survival relative to low (<24%) or high (≥24%) proliferative index.*

and could contribute to drug resistance. Three studies have shown that elevated levels of mdr-1 are associated with an adverse outcome in osteosarcoma.[54–56]

In summary, this study suggests that cell proliferation, as indicated by Ki-67 expression detected by immunohistochemical staining with MIB-1 antibody in formalin fixed paraffin-embedded sections, can be quantified either by manual cell counts using an ocular grid or by determining the percent immunopositive nuclear area by image morphometry. An increased proliferative index, as determined in biopsies of high grade osteosarcoma by either quantification method, does not appear to be prognostic for outcome in this retrospective study. Assessment of tumor cells for the presence of changes in tumor suppressor genes or cell cycle regulators may further enhance our ability to predict prognosis, but this requires further investigation.

### Acknowledgements

We would like to thank Lori Cutler and Isabelle Schell for their excellent secretarial assistance.

### References

1 Link MP, Goorin AM, Horowitz M, Meyer WH, Belasco J, Baker A, Ayala A, Shuster J. Adjuvant chemotherapy of high-grade osteosarcoma of the extremity. Updated results of the multi-institutional osteosarcoma study. *Clin Orthop Rel Res* 1991;270:8–14.

2 Glasser DB, Lane JM, Huvos AG, Marcove RC, Rosen G. Survival, prognosis, and therapeutic response in osteogenic sarcoma. *Cancer* 1992;69:698–708.

3 Unni KK. *Dahlin's Bone Tumors. General Aspects and Data on 11,087 Cases*, 5th edn. Philadelphia: Lippincott-Raven, 1996:143–83.

4 Petrilli AD, Gentil FC, Epelman S, Lopes LF, Bianchi A, Lopes A, De Assis Figueiredo MT, Marques E, De Bellis N, Consentino E, Prospero D, De Camargo OP, Oliveira NR, Franco E, Jaffe N. Increased survival, limb preservation, and prognostic factors for osteosarcoma. *Cancer* 1991;68:733–37.

5 Davis AM, Bell RS, Goodwin PJ. Prognostic factors in 'classical' osteosarcoma: a critical appraisal. *J Clin Oncol* 1994;12:423–31.

6 Raymond AK, Chawla SP, Carrasco H, Ayala AG, Fanning CV, Grice B, Armen T, Plager C, Papadopoulos NEJ, Edeiken J, Wallace S, Jaffe N, Murray JA, Benjamin RS. Osteosarcoma chemotherapy effect: a prognostic factor. *Sem Diagn Pathol* 1987;4:212–36.

7 Eastman A. Activation of programmed cell death by anticancer agents: cisplatin as a model system. *Cancer Cells* 1990;2:275–80.

8 Hickman JA. Apoptosis induced by anticancer drugs. *Cancer Metastasis Rev* 1992;11:121–39.

9 Lowe SW, Ruley HE, Jacks T, Housman DE. p53-dependent apoptosis modulates the cytotoxicity of anticancer agents. *Cell* 1993;74:957–67.

10 Lowe SW, Bodis S, McClatchey A, Remington L, Ruley HE, Fisher DE, Housman DE, Jacks T. p53 status and the efficacy of cancer therapy in vivo. *Science* 1994;266:807–10.

11 Fan S, El-Deiry WS, Bae I, Freeman J, Jondle D, Bhatia K, Fornace AJ Jr., Magrath I, Kohn KW, O'Connor PM. p53 gene mutations are associated with decreased sensitivity of human lymphoma cells to DNA damaging agents. *Cancer Res* 1994;54:5824–30.

12 Aas T, Børresen AL, Geisler S, Smith-Sorensen B, Johnsen H, Varhaug JE, Akslen LA, Lønning PE. Specific p53 mutations are associated with de novo resistance to doxorubicin in breast cancer patients. *Nature Medicine* 1996;2:811–13.

13 Schmitt CA, Lowe SW. Apoptosis and therapy. *J Pathol* 1999;187:127–37.

14 Meikrantz W, Schlegel R. Apoptosis and the cell cycle. *J Cell Biochem* 1995;58:160–74.

15 King KL, Cidlowski JA. Cell cycle and apoptosis: common pathways to life and death. *J Cell Biochem* 1995;58:175–80.

16 Choong PFM, Akerman M, Willén H, Andersson C, Gustafson P, Alvegòrd T, Rydholm A. Expression of proliferating cell nuclear antigen (PCNA) and Ki-67 in soft tissue sarcoma. Is prognostic significance histotype-specific? *APMIS* 1995;103:797–805.

17 Jensen V, Sorensen FB, Bentzen SM, Ladekarl M, Nielsen OS, Keller J, Jensen OM. Proliferative activity (MIB-1 index) is an independent prognostic parameter in patients with high-grade soft tissue sarcomas of subtypes other than malignant fibrous histiocytomas: a retrospective immunohistochemical study including 216 soft tissue sarcomas. *Histopathology* 1998;32:536–46.

18 Heslin MJ, Cordon-Cardo C, Lewis JJ, Woodruff JM, Brennan MF. Ki-67 detected by MIB-1 predicts distant metastasis and tumor mortality in primary, high grade extremity soft tissue sarcoma. *Cancer* 1998;83:490–97.

19 Kirkegaard LJ, DeRose PB, Yao B, Cohen C. Image cytometric measurement of nuclear proliferation markers (MIB-1, PCNA) in astrocytomas. *Am J Clin Pathol* 1998;109:69–74.
20 Thor AD, Liu S, Moore DH II, Edgerton SM. Comparison of mitotic index, in vitro bromodeoxyuridine labeling, and MIB-1 assays to quantitate proliferation in breast cancer. *J Clin Oncol* 1999;17:470–77.
21 Elias JM. *Immunohistopathology. A Practical Approach to Diagnosis*, 5th edn. Chicago: ASCP Press, 1999:9.
22 Kaplan EL, Meier P. Nonparametric estimation from incomplete observations. *J Amer Stat Assoc* 1958;53:457–81.
23 Layfield LJ, Saria EA, Berchuck A, Dodge RK, Thompson JK, Conlon DH, Kerns B-JM. Prognostic value of MIB-1 in advanced ovarian carcinoma as determined using automated immunohistochemistry and quantitative image analysis. *J Surg Oncol* 1997;66:230–37.
24 Biesterfeld S, Kluppel D, Koch R, Schneider S, Steinhagen G, Mihalcea AM, Schroder W. Rapid and prognostically valid quantification of immunohistochemical reactions by immunohistometry of the most positive tumour focus. A prospective follow-up study on breast cancer using antibodies against MIB-1, PCNA, ER and PR. *J Pathol* 1998;185:25–31.
25 Minimo C, McCue PA, Pindzola A, Brennan J, Bibbo M. Role of computed quantitation of immunohistochemical staining of Ki-67 antigen in diagnosing ampullary lesions. *Anal Quant Cytol Histol* 1996;18:400–4.
26 Caulet S, Lesty C, Raphael M, Binet JL, Diebold J. Quantitative study of Ki-67 antibody staining in non-Hodgkin's lymphomas using image analysis. *Anal Quant Cytol Histol* 1991;13:279–87.
27 Gerdes J, Li L, Schlueter C, Duchrow M, Wohlenberg C, Gerlach C, Stahmer I, Kloth S, Brandt E, Flad H-D. Immunobiochemical and molecular biologic characterization of the cell proliferation-associated nuclear antigen that is defined by monoclonal antibody Ki-67. *Am J Pathol* 1991;138:867–73.
28 Risio M. Methodological aspects of using immunohistochemical cell proliferation biomarkers in colorectal carcinoma chemoprevention. *J Cell Biochem* 1994;19:61–7.
29 Hendricks JB, Wilkinson EJ. Quality control considerations for Ki-67 detection and quantitation in paraffin-embedded tissue. *J Cell Biochem* 1994;19:105–10.
30 Dictor M, Ehinger M, Mertens F, Akervall J, Wennerberg J. Abnormal cell cycle regulation in malignancy. *Am J Clin Pathol* 1999;112:S40–S52.
31 Bubendorf L, Sauter G, Moch H, Schmid HP, Gasser TC, Jordan P, Mihatsch MJ. Ki67 labelling index: an independent predictor of progression in prostate cancer treated by radical prostatectomy. *J Pathol* 1996;178:437–41.
32 Valente G, Giusti U, Kerim S, Gabriele P, Motta M, Ragona R, Navone R, Palestro G. High prognostic impact of growth fraction parameters in advanced stage laryngeal squamous cell carcinoma. *Oncol Rep* 1999;6:289–93.
33 Mochen C, Giardini R, Costa A, Silvestrini R. MIB-1 and S-phase cell fraction predict survival in non-Hodgkin's lymphomas. *Cell Prolif* 1997;30:37–47.
34 Nawa G, Ueda T, Mori S, Yoshikawa H, Fukuda H, Ishiguro S, Funai H, Uchida A. Prognostic significance of Ki67 (MIB1) proliferation index and p53 overexpression in chondrosarcomas. *Int J Cancer* 1996;69:86–91.
35 Sallinen PK, Haapasalo HK, Visakorpi T, Helen PT, Rantala IS, Isola JJ, Helin HJ. Prognostication of astrocytoma patient survival by Ki-67 (MIB-1), PCNA, and S-phase fraction using archival paraffin-embedded samples. *J Pathol* 1994;174:275–82.
36 Shivji KK, Kenny MK, Wood RD. Proliferating cell nuclear antigen is required for DNA excision repair. *Cell* 1992;69:367–74.
37 Woosley JT. Measuring cell proliferation. *Arch Pathol Lab Med* 1991;115:555–7.
38 Hitchcock CL. Ki-67 staining as a means to simplify analysis of tumor cell proliferation. *Am J Clin Pathol* 1991;96:444–6.
39 Rudolph P, Peters J, Lorenz D, Schmidt D, Parwaresch R. Correlation between mitotic and Ki-67 labeling indices in paraffin-embedded carcinoma specimens. *Hum Pathol* 1998;29:1216–22.
40 Sahin AA, Ro JY, El-Naggar AK, Wilson PL, Teague K, Blick M, Ayala AG. Tumor proliferative fraction in solid malignant neoplasms. *Am J Clin Pathol* 1991;96:512–9.
41 Linden MD, El-Naggar AK, Nathanson SD, Jacobson G, Zarbo RJ. Lack of correlation between flow cytometric and immunohistologic proliferation measurements of tumors. *Mod Pathol* 1996;9:682–9.
42 Irazusta SP, Vassallo J, Magna LA, Metze K, Trevisan M. The value of PCNA and AgNOR staining in endoscopic biopsies of gastric mucosa. *Pathol Res Prac* 1998;194:33–9.
43 Soomro IN, Whimster WF. Growth fraction in lung tumours determined by Ki67 immunostaining and comparison with AgNOR scores. *J Pathol* 1990;162:217–22.
44 Ohno T, Tanaka T, Takeuchi S, Matsunaga T, Mori H. Nucleolar organizer regions in bone tumors. *Clin Orthop Rel Res* 1991;272:287–91.
45 Park H-R, Park Y-K. Expression of p53 protein, PCNA, and Ki-67 in osteosarcomas of bone. *J Korean Med Sci* 1995;10:360–7.
46 Scotlandi K, Serra M, Manara C, Maurici D, Benini S, Nini G, Campanacci M, Baldini N. Clinical relevance of Ki-67 expression in bone tumors. *Cancer* 1995;75:806–14.
47 Molendini L, Benassi MS, Magagnoli G, Merli M, Sollazzo MR, Ragazzini P, Gamberi G, Ferrari C, Balladelli A, Bacchini P, Picci P. Prognostic significance of cyclin expression in human osteosarcoma. *Int J Oncol* 1998;12:1007–11.
48 Jensen ML, Schumacher B, Jensen OM, Nielsen OS, Keller J. Extraskeletal osteosarcomas. A clinicopathologic study of 25 cases. *Am J Surg Pathol* 1998;22:588–94.
49 Viberti L, Papotti M, Abbona GC, Celano A, Filosso PL, Bussolati G. Value of Ki-67 immunostaining in preoperative biopsies of carcinomas of the lung. *Hum Pathol* 1997;28:189–92.
50 Bubendorf L, Tapia C, Gasser TC, Casella R, Grunder B, Moch H, Mihatsch MJ, Sauter G. Ki67 labeling index in core needle biopsies independently predicts tumor-specific survival in prostate cancer. *Hum Pathol* 1998;29:949–54.
51 van Oijen MGCT, Medema RH, Slootweg PJ, Rijksen G. Positivity of the proliferation marker Ki-67 in noncycling cells. *Am J Clin Pathol* 1998;110:24–31.
52 Benassi MS, Molendini L, Gamberi G, Sollazzo MR, Ragazzini P, Merli M, Magagnoli G, Sangiorgi L, Bacchini P, Bertoni F, Picci P. Altered $G_1$ phase regulation in osteosarcoma. *Int J Cancer* 1997;74:518–22.
53 Chan HSL, Grogan TM, Haddad G, Hipfner DR, Deeley RG, Cole SPC. Standardization of a single-cell assay for sensitive detection of multidrug resistance protein expression in normal and malignant cells in archival clinical samples. *J Lab Clin Med* 1997;130:297–306.

54 Baldini N, Scotlandi K, Barbanti-Brodano B, Manara MC, Maurici D, Bacci G, Bertoni F, Picci P, Sottili S, Campanacci M, Serra M. Expression of p-glycoprotein in high-grade osteosarcoma in relation to clinical outcome. *N Engl J Med* 1995;333:1380–5.

55 Radig K, Hackel C, Herting J, Oda Y, Mittler U, Neumann W, Roessner A. Expression of p-glycoprotein in high grade osteosarcomas with special emphasis on chondroblastic subtype. *Gen Diagn Pathol* 1996/97;142:139–45.

56 Serra M, Maurici D, Scotlandi K, Barbanti-Brodano G, Manara MC, Benini S, Picci P, Bertoni F, Bacci G, Sottili S, Baldini N. Relationship between p-glycoprotein expression and p53 status in high-grade osteosarcoma. *Int J Oncol* 1999;14:301–307.

57 *AJCC Cancer Staging Manual*, 5th edn. Bone. Philadelphia: Lippincott-Raven, 1997;144.
